# Supplementary material for: The Hsf1-sHsp cascade has pan-antiviral activity in mosquito cells
Source: Commun Biol. 2025 Jan 25;8:123. doi: 10.1038/s42003-024-07435-4 (PMC11762766; doi:10.1038/s42003-024-07435-4)
Supplement: Supplementary file 2 — Supplementary information [file 42003_2024_7435_MOESM2_ESM.pdf]

Supplementary information for:

The Hsf1-sHsp cascade has pan-antiviral activity in mosquito cells

Jieqiong Qu[#], Michelle Schinkel[#], Lisa Chiggiato, Samara Rosendo Machado, Gijs J. Overheul, Pascal Miesen, Ronald P. van Rij *

Department of Medical Microbiology, Radboud University Medical Center, P.O. Box 9101, 6500 HB Nijmegen, The Netherlands

[#] These authors contributed equally to this study

* For correspondence: Ronald.vanRij@radboudumc.nl

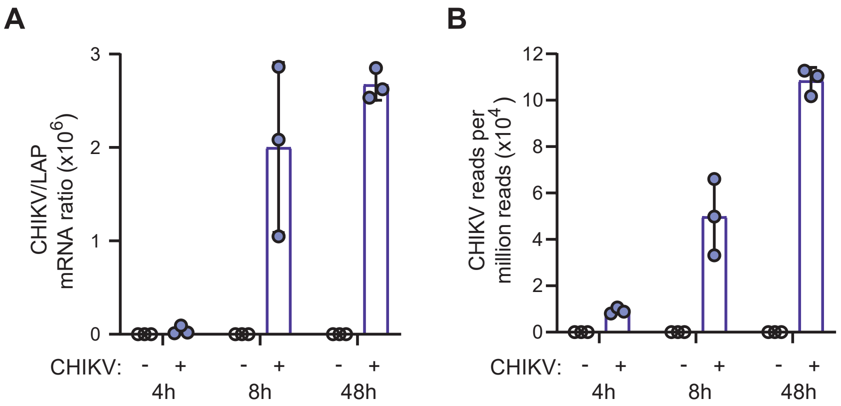

Supplementary Figure 1. Chikungunya virus infection dynamics in *Ae. aegypti* cells. (A–B) CHIKV RNA levels quantified by RT-qPCR (A) and RNA-seq (B) in mock (-) or CHIKV (MOI = 5, +) infected Aag2 cells at 4, 8, and 48 h post infection. Viral RNA levels were expressed relative to the LAP housekeeping gene in (A). RT-qPCR and RNA-seq was performed in biological triplicates shown as dots, with bars indicating mean ±SD.

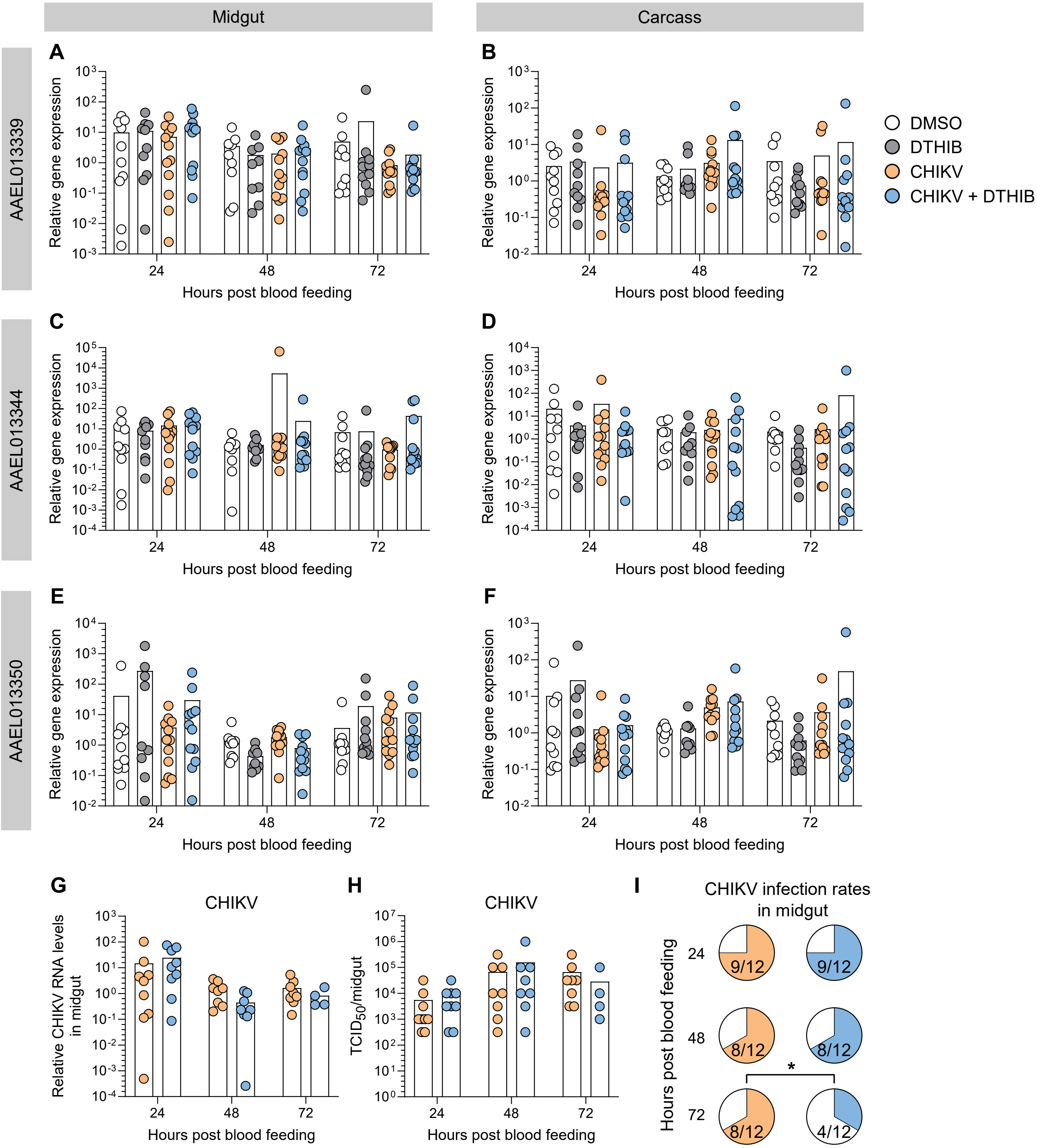

Supplementary Figure 2. Small molecule compound Direct Targeted Hsf1 InhiBitor (DTHIB) does not modulate sHsp gene expression ***in vivo*****.** (A–F) mRNA expression of representative sHsp genes in Aedes aegypti midgut (A, C, E) or carcass (B, D, F) at 1, 2 and 3 days after a blood meal containing CHIKV (1.7×10^8 TCID50/ml) and/or 10 µM DTHIB, as indicated. DMSO was used as a negative control. (G–I) CHIKV RNA levels (G), titers (H), and infection rates (I) in Aedes aegypti midguts at 1, 2 and 3 days after an infectious CHIKV blood meal, supplemented with 10 µM DTHIB or DMSO as a control. In (A–H), data from individual mosquitoes are shown, with bars indicating the mean (n = 9–12 mosquitoes per group). Viral and cellular RNA was quantified by RT-qPCR. Differences in infection rates were assessed using Chi-squared tests (*$p < 0.05$).
Expression of sHsp genes was not significantly different (*t*-tests) between experimental groups at all timepoints (A–F), suggesting that DTHIB treatment does not modulate sHsp expression *in vivo* or that it does not reach effective concentrations when provided via a blood meal. Accordingly, viral RNA levels and titers were not significantly different (Mann-Whitney U tests) between DTHB and control treated mosquitoes (G–H). Although the infection rates in the midgut (the proportion of virus infected mosquitoes) were significantly reduced at 3 days post infection in the presence of DTHIB, this was not consistently seen at earlier time points (I). Other hsf1 modulating compounds KRIBB11 and hsf1a were poorly soluble in blood and were therefore not used for *in vivo* experiments. Lack of robust sHsp gene induction by CHIKV infection (A–F) is likely due to the relatively late time point analyzed and/or to a non-synchronous infection, compared to the high MOI infection used in Aag2 cells (Fig. 1). The role of the Hsf1-sHsp cascade for antiviral defense *in vivo* thus remains to be established, which requires the development of novel methods to inactivate Hsf1 function.
