# Supplementary material for: The Hsf1-sHsp cascade has pan-antiviral activity in mosquito cells
Source: Commun Biol. 2025 Jan 25;8:123. doi: 10.1038/s42003-024-07435-4 (PMC11762766; doi:10.1038/s42003-024-07435-4)
Supplement: Supplementary file 4 — Description of Additional Supplementary File [file 42003_2024_7435_MOESM4_ESM.pdf]

Description Of Additional Supplementary File

File name: Supplementary Data 1
Description: Differentially expressed genes (DEgenes) and FPKM values upon CHIKV infection of Aag2 cells at different time points.

File name: Supplementary Data 2
Description: Source data for the graphs in the paper.
